# Impaired Pancreatic β-Cell Function in Critically Ill Children

**DOI:** 10.3389/fped.2021.603361

**Published:** 2021-03-18

**Authors:** Shereen A. Mohamed, Nora E. Badawi, Hoiyda A. AbdelRasol, Hossam M. AbdelAziz, Nirvana A. Khalaf, Remon M. Yousef

**Affiliations:** ^1^Pediatric Department, Kasr Al-Ainy School of Medicine, Cairo University, Cairo, Egypt; ^2^Clinical Pathology Department, Faculty of Medicine, Fayoum University, Fayoum, Egypt; ^3^Clinical Pathology Unit, Research Institute of Ophthalmology, Giza, Egypt; ^4^Pediatric Department, Faculty of Medicine, Fayoum University, Fayoum, Egypt

**Keywords:** β cell dysfunction, PRISM III score, hyperglycemia, pediatric ICU, HOMA-β

## Abstract

Critical illness hyperglycemia (CIH) is common in the pediatric intensive care unit (PICU). Increased glucose production, insulin resistance (IR), and pancreatic β-cell dysfunction are responsible mechanisms. We aimed to investigate β-cell function in the PICU and to uncover its relation to clinical and laboratory variables and ICU mortality. We prospectively recruited 91 children. Pancreatic β-cell function was assessed by using a homeostasis model assessment (HOMA)-β. Patients with β-cell function <40.0% had significantly higher Pediatric Risk of Mortality III (PRISM III) scores, higher rates of a positive C-reactive protein (CRP), lower IR, and a longer hospital stay. The patients with 40–80% β-cell function had the highest IR. Intermediate IR was found when the β-cell function was >80%. ICU survivors had better β-cell function than ICU non-survivors. A multivariate logistic regression analysis revealed that higher PRISM III score and HOMA-β <80.0% were significant predictors of mortality. In conclusion, β-cell dysfunction is prevalent among PICU patients and influences patient morbidity and mortality.

## Introduction

The management of glucose and insulin homeostasis in critically ill children is a complex issue. Hyperglycemia and its related complications remain the most challenging endocrinological problem in the context of stress response (1). Since the publication of the Leuven study in 2001, the role of tight glycemic control in the management of critically ill children and adults continues to be a matter of argument (2). However, recent evidence has undermined the value of tight glycemic control in pediatric intensive care unit (PICU). While it did not result in a reduced mortality, it was associated with an increased risk of hypoglycemia (3). Hypoglycemia, in turn, is significantly associated with a worse short-term outcome (4).

While several studies have investigated the relation between several glucose and insulin homeostasis parameters and the outcome in critically ill children (5–10), a few works discussed β-cell function in this population.

Conventionally, stress hyperglycemia in ICU patients was explained by increased hepatic production of glucose by gluconeogenesis and glycogenolysis and the development of insulin resistance (IR) (11). Nevertheless, insulin deficiency due to pancreatic β-cell dysfunction was also blamed. In this condition, β-cell dysfunction was attributed to cellular over exhaustion due to the requirement of huge insulin demand to overcome IR (12). Moreover, a recent study provided evidence that interlukin-1β-mediated apoptosis of pancreatic β cell is another mechanism for acute illness hyperglycemia (13). In the present study, we sought to investigate the presence of stress hyperglycemia, β-cell function, and IR in children admitted to the PICU for various conditions and to uncover the clinical and laboratory variables [such as Pediatric Risk of Mortality III (PRISM III) score and C-reactive protein (CRP)] that may accompany this condition. We hypothesized that β-cell dysfunction could be associated with a greater risk for mortality in the ICU setting.

## Patients and Methods

In this prospective study, we recruited all eligible children admitted to the PICU of Fayoum University Hospital for an 8-month period between December 2019 and July 2020. The study protocol was approved by the Research Ethical Committees of Fayoum University and was conducted in accordance with the ethical principles described in the Declaration of Helsinki. Informed consent was received from the guardians of all the participants.

**Figure 1:**
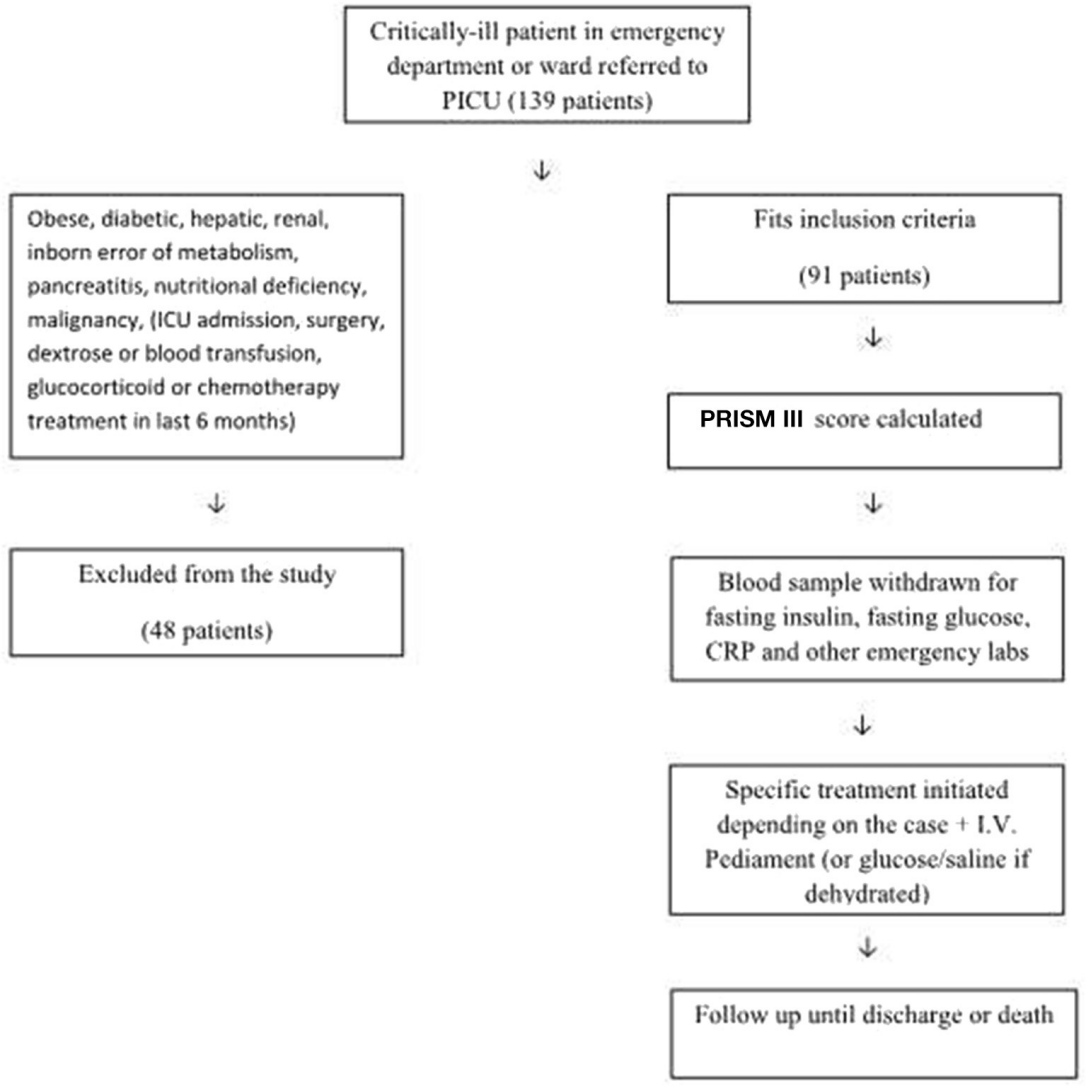
Flow diagram for recruitment and assessment of β-cell function in critically-ill children.

Figure 1 demonstrates the flow diagram for sorting the patients for eligibility. All patients admitted to PICU, who were referred from the pediatric ward, emergency department, or operating theater (after the assessment and approval by the ICU consultant), aged 1 month to 14 years, were included if they had been previously healthy. Patients were eliminated from the study if they were obese, had diabetes, previous liver or renal diseases, inborn errors of carbohydrate metabolism, known or suspected pancreatitis, malignant tumors, marked nutritional deficiency (severe malnutrition = very low weight/height, i.e., below −3 Z-scores from the median WHO growth standards, evident from visible severe wasting, or by the presence of nutritional edema) (14) or if they had been subjected to major surgery, blood transfusions, dextrose infusion, glucocorticoid administration, or chemotherapy in the 6 months prior to the PICU admission or if they had been fed within 6 h before the admission.

Patients had received either no fluids or were on normal saline infusion prior to the PICU admission. After sampling, they were placed on intravenous fluids (pediament in case of no dehydration and glucose/saline fluids in case of dehydration) pending test results. Relevant drug therapy was instituted at this point depending on their condition.

Severity of critical illness was evaluated by using PRISM III score (15).

Upon admission to the PICU, basic demographic and clinical data were documented and complete standard laboratory workup was performed within 24 h. Blood samples were taken on admission by the phlebotomist after venous cannulation and before introducing any form of treatment (catecholamines, glucocorticoids, or any other drugs that might affect blood glucose levels). Six-hour fasting blood sugar was assayed by using SYNCHRON CX-9 Autoanalyzer (Beckman Instrument Corporation, CA, USA). Fasting insulin was measured by using immunometric chemiluminescent assay on IMMULITE Autoanalyzer (Siemens Medical Solution Diagnostics, LA, USA).

To measure pancreatic β-cell function and IR, we used a homeostasis model assessment (HOMA)-1. HOMA-1 IR is widely used and has been validated against the glucose clamp technique (the gold standard method to assess IR) (16).

Stress hyperglycemia was considered to exist when fasting blood glucose was >125 mg/dl, and impaired glucose tolerance when fasting blood glucose measured 100–125 mg/dl (17).

Pancreatic β-cell function was assessed by using HOMA-β according to the formula: HOMA-β = fasting insulin (mU/ml) × 20/[fasting glucose (mmol/ml) −3.5], where a normal β cell is 100%. Since there are no clear cutoff points for the evaluation of the degree of HOMA-β dysfunction, we classified patients into three groups according to the β-cell function (GI: <40%, GII: 40–80%, and GIII: >80%) after the study of Liu et al. (18).

Insulin resistance was evaluated by using the HOMA-IR model according to the formula: HOMA-IR = fasting insulin (mU/ml) × fasting glucose (mmol/ml)/22.5 (19), where normal IR is 1.

Reduced glucose/insulin ratio <7 reflects an abnormal (reduced) insulin sensitivity (20).

Due to normal individual differences in insulin sensitivity, one of the limitations of the HOMA model is that β-cell function cannot be viewed in isolation. For this reason, glucose and insulin levels must be measured simultaneously in the same blood sample (21).

C-reactive protein was measured by using immunoturbidimetry and was considered negative at <6 mg/L

The outcome measures included mechanical ventilation duration, the use of inotropic drug support, PICU length of stay, and PICU mortality.

A sample size of 90 patients was assessed based on the study of El-Sherbini et al. (22) where β-cell dysfunction was reported in 43% of critically ill children using epiinfo 7.2.2.6 with a confidence level of 95% (alpha error of 5%) and an acceptable margin of error of 10%. Statistical analysis was performed by using SPSS 15.0 (IBM, Chicago, IL, USA). Data are presented as frequency and percent, mean ± SD or median and interquartile range (IQR). Another issue is that HOMA estimates of β-cell function and insulin sensitivity are usually not normally distributed. For this reason, we measured data for normality, logarithmically transformed it and reported it as geometric mean (21). Comparative statistics were performed by using the Mann–Whitney *U*-test, the Kruskal–Wallis test, the chi-squared test, or the Fisher's exact test as appropriate. A forward stepwise multiple logistic analysis was applied to identify the HOMA-β% (<80 vs. >80) as a predictor for mortality after adjustment for age, CRP, and PRISM III. PRISM III was included in the model because it was identified as a statistically significant predictor in a univariate analysis. Also, the multivariate model was adjusted for age and CRP although they were not statistically significant by univariate logistic analysis (18, 22, 23) *p* < 0.05 was considered significant.

## Results

A total of 139 children were eligible, of whom 48 were excluded for the following reasons: 15 had diabetic ketoacidosis, 7 had received corticosteroids, 6 had kidney disease, 5 were severely malnourished, 5 had received glucose infusion, 4 had received blood transfusions, 3 had been recently fed, 2 had liver cell failure, and 1 was obese. The remaining 91 subjects met the inclusion criteria. Of the total 91 subjects, 35 (38.5%) had stress hyperglycemia and 23 (25%) had an impaired glucose tolerance. About 20 patients had a β-cell function>100% (up to 7.4 times of normal) and IR>1, which were capable of maintaining normoglycemia in these patients. Table 1 compares the clinical data and outcome parameters in the studied patients classified according to their % β-cell function. Group I patients (β-cell function <40%, *n* = 57, i.e., 62.6% of the study group) had significantly higher fasting blood glucose levels and lower fasting insulin levels resulting in the highest glucose/insulin ratio. The lowest HOMA-IR and highest PRISM scores were also observed in this group. A significantly higher percentage of this group had positive CRPs (57.9% compared to 41.7% in Group II and 13.6% in Group III (% β-cell function >80, *n* = 22, i.e., 24.1% of the study group) *p* = 0.002.

**Table 1:** Clinical data, laboratory data, and outcome in the studied patients classified according to β-cell function (*N* = 91).

		**Group 1 HOMA-β% <40 *n* = 57**	**Group 11 HOMA-β% 40–80 *n* = 12**	**Group 111 HOMA-β% >80 *n* = 22**	
Age (months) median (IQR)		11 (4–24)	4 (3–11.5)	6 (2–36)	0.191
BMI Z score median (IQR)		−1.3 (−2.0–0.8)	1.25 (0.70–2.0)	−1.7 (−2.0–0.0)	**0.007**
**Sex** ***n*** **(%)**					
Male		32 (56.1)	3 (25.0)	12 (54.5)	0.139
Female		25 (43.9)	9 (75.0)	10 (45.5)	
**Diagnosis** ***n*** **(%)**					
Respiratory		19 (33.3)	8 (66.7)	2 (9.1)	**0.003**
CVS		8 (14.0)	2 (16.7)	–	0.16
CNS		7 (12.3)	1 (8.3)	6 (27.3)	0.195
GIT & hepatic		2 (3.5)	–	–	0.54
Sepsis		12 (21.1)	1 (8.3)	3 (13.6)	0.492
Surgical		–	–	10 (45.5)	** <0.001**
Metabolic		4 (7.0)	–	1 (4.5)	0.61
Hematological		2 (3.5)	–	–	0.54
Renal		3 (5.3)	–	–	0.4
PRISM score median (IQR)		5 (3–8)	3 (3–4.75)	3 (0–4.5)	**0.015**
Fasting blood glucose (mg/dl) median (IQR)		137.0 (108.0–186.5)	118.5 (115.3–136.0)	73.5 (68.0–100.5)	** <0.001**
Fasting insulin (mIU/L) median (IQR)		1.93 (0.5–4.2)	7.65 (6.35–10.75)	10.3 (10.3–16.3)	** <0.001**
Glucose/insulin ratio (median (IQR)		73.8 (37–238.8)	16.2 (12.3–18.2)	6.6 (6.2–7.1)	** <0.001**
HOMA-β (%)	median (IQR)	12.56 (4.37–18.9)	48.36 (43.66–57.33)	531.34 (192.52–741.60)	** <0.001**
Log HOMA-β (%)	Geometric mean (95% CI)	8.14 (6.05–10.9)	50.8 (45.2–57.1)	349 (242–504)	** <0.001**
HOMA-IR	median (IQR)	0.66 (0.11–1.51)	2.09 (1.64–3.17)	1.57 (1.56–3.72)	** <0.001**
Log Homa-IR	Geometric mean (95% CI)	0.55 (0.38–0.85)	2.13 (1.55–2.93)	2.59 (178–3.79)	** <0.001**
CRP (Positive) *n* (%)		33 (57.9)	5 (41.7)	3 (13.6)	**0.002**
Inotropic use *n* (%)		56 (98.2)	11 (91.7)	22 (100.0)	0.266
PICU stay (days) median (IQR)		4 (3–6)	5.5 (4–10)	3.5 (3–7)	0.141
Hospital stay (days) median (IQR)		5 (4–7)	9 (5.5–10)	5 (3–7)	**0.040**
MV duration (days) median (IQR)		1 (0–4)	0 (0–4)	–	**0.020**
Mortality *n* (%)		15 (26.3)	3 (25.0)	1 (4.5)	0.096

*MV, mechanical ventilation; PRISM, Pediatric risk of mortality score; BMI, body mass index; CVS, cardiovascular system; CNS, central nervous system; GIT, gastrointestinal tract*.

*HOMA-IR, Homeostatic model assessment-Insulin resistance; IQR, interquartile range*.

*CI, confidence interval; C-Reactive protein (CRP) was considered negative if <5 mg/L*.

*Normal fasting glucose <100 mg/dL Impaired glucose tolerance: 100–125 mg/dL*.

*A p < 0.05 was significant*.

*Significant values are indicated in bold*.

Group II had intermediate levels of % β-cell function (40–80%, *n* = 12, i.e., 13.1% of the study group). This group, with significantly higher BMI Z-scores, also had higher HOMA-IR values than the other two groups as well as the longest duration of hospital and PICU stays.

Table 2 compares PICU survivors, *n* = 72 (79.1%) and non-survivors, *n* = 19 (20.9%). Survivors had a significantly lower PRISM score (*p* = 0.039) and mechanical ventilation duration (*p* = 0.012), higher fasting insulin levels (*p* = 0.03), and lower fasting glucose/insulin ratio (*p* = 0.036). HOMA-β% was not statistically higher in the survivors with a *p*-value of 0.05. There was no difference in the survival between males and females.

**Table 2:** Comparison between survivors and non-survivors with regard to clinical data and outcome parameters (*N* = 91).

**Variables**		**Mortality**		***P*-value**
		**Survivors (*N* = 72)**	**Non-survivors (*N* = 19)**	
Age (months), median (IQR)		7.5 (3.6–30)	10 (3–20)	0.887
**Sex**, ***n*** **(%)**				
Male		39 (54.2%)	8 (42.1%)	0.349
Female		33 (45.8%)	11 (57.9%)	
BMI Z score		−1.05 (−2.0–1.15)	−0.25 (2.0–1.0)	0.692
**Diagnoses**, ***n*** **(%)**				
Respiratory		23 (31.9%)	6 (31.6%)	0.976
CVS		7 (9.7%)	3 (15.8%)	0.452
CNS		11 (15.3%)	3 (15.8%)	0.956
GIT & hepatic		1 (1.4%)	1 (5.3%)	0.306
Sepsis		12 (16.7%)	4 (21.1%)	0.655
Surgical		10 (13.9%)	–	0.09
Metabolic		5 (6.9%)	–	0.24
Hematological		1 (1.4%)	1 (5.3%)	0.306
Renal		2 (2.8%)	1 (5.3%)	0.589
PRISM, median (IQR)		3 (2–6)	6 (3–8)	**0.039**
Fasting insulin (mIU/L/), median (IQR)		5.95 (1.77–10.3)	1.9 (0.53–5)	**0.030**
HOMA-β %	median (IQR)	25.97 (7.05–105.66)	14.52 (6.34–22.25)	0.050
Log HOMA- β (%)	Geometric mean (95% CI)	31.37 (19.83–49.62)	12.08 (6.38–22.88)	**0.048**
HOMA-IR	median (IQR)	1.56 (0.64–2.43)	0.68 (0.22–2.24)	0.105
Log Homa-IR	Geometric mean (95% CI)	1.1 (0.76–1.58)	0.57 (0.28–1.16)	0.101
CRP (Positive) *n* (%)		33 (45.8%)	8 (42.1%)	0.771
Inotropic use, *n* (%)		71 (98.6%)	18 (94.7%)	0.376
PICU stay (days), median (IQR)		4 (3–6)	5 (3–10)	0.226
Hospital stay (days), median (IQR)		5.5 (4–7)	5 (4–10)	0.425
MV (days), median (IQR)	0 (0–4)		2 (1–3)	**0.012**

*MV, mechanical ventilation; PRISM, Pediatric risk of mortality score; CVS, cardiovascular system; CNS, central nervous system; GIT, gastrointestinal tract; IQR, interquartile range*.

*CI, confidence interval; HOMA-IR, Homeostatic model assessment-Insulin resistance; CRP, C-reactive protein (N < 5 mg/L)*.

*Significant values are indicated in bold*.

A multivariate logistic regression analysis revealed that higher PRISM score and HOMA-β% < 80.0% were the only significant predictors of mortality (Table 3).

**Table 3:** Predictors of mortality in the studied patients (*N* = 91).

	**Univariate**			**Multivariate**		
	**OR**	**95% CI**	***P*-value**	**OR**	**95% CI**	***P*-value**
HOMA β% (<80 vs. >80)	7.412	(0.929–59.138)	0.059	**8.574**	**(1.012–72.639)**	**0.049**
Age	1.003	(0.991–1.016)	0.596	0.999	(0.986–1.013)	0.939
CRP	1.163	(0.419–3.233)	0.772	2.472	(0.777–7.862)	0.125
PRISM-III	1.166	(1.019–1.333)	0.025	1.169	(1.011–1.351)	**0.035**

*HOMA-β% Homeostatic model assessment- β-cell function; CRP, C-reactive protein (N < 5); PRISM, Pediatric risk of mortality score*.

*Significant values are indicated in bold*.

**Figure 2:**
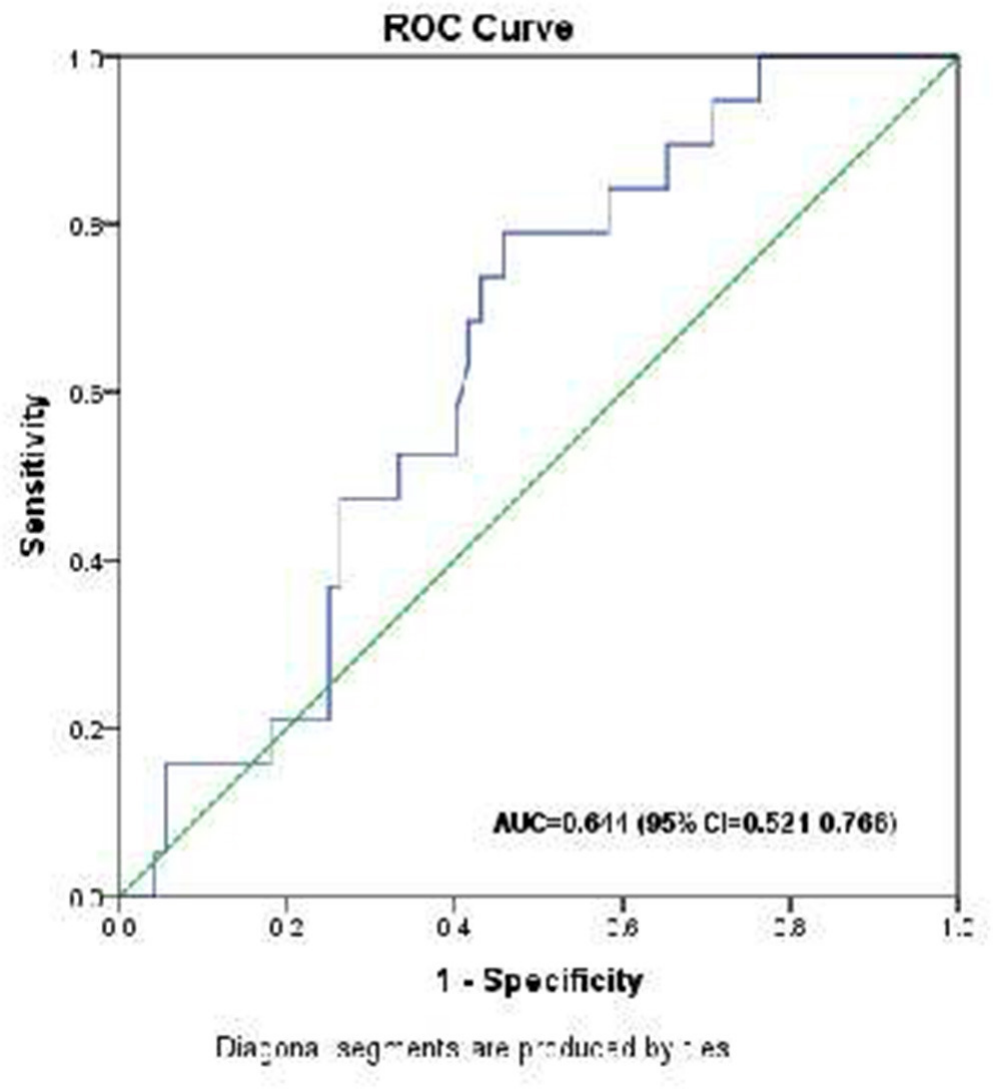
Receiver operating characteristic (ROC) curve analysis for HOMA-β% in the prediction of mortality in critically ill children.

The optimal cutoff value of HOMA-β% for mortality prediction was 22.52 producing a sensitivity of 78.9% and specificity of 54.2% [(positive predictive value (PPV) = 31.3% and negative predictive value (NPV) = 90.7% with Area Under the Curve (AUC) = 0.702 (95% CI 0.521–0.766), Youden index = 134.1] (Figure 2).

## Discussion

The present study documented that β-cell dysfunction is prevalent in critically ill children. These findings add evidence to previously published studies. In 2009, Preissig and Rigby (23) challenged the idea that critical illness hyperglycemia (CIH) is caused by an increased IR and reported a significant deterioration of β-cell function in children with CIH. However, instead of the HOMA-β model, they used C-peptide to assess β-cell function. In a particular group of critically ill children with meningococcal sepsis and septic shock, Verhoeven et al. (24) used a HOMA-β model of <50.0% to diagnose β-cell dysfunction in 38% of the studied children. Later, the study of Hacihamdioglu et al. (25) followed hyperglycemic critically ill children until hyperglycemia resolved and found significantly higher levels of β-cell dysfunction as assessed by the HOMA-β model in the hyperglycemic periods in comparison to the euglycemic periods.

In agreement with our conclusions, the study of Liu et al. (18) on 1,146 critically ill children classified according to their β-cell dysfunction level found a significant and continuous deterioration of disease severity (PRISM) score as β-cell dysfunction progressed.

In our study, there was a significant association between a reduced β-cell function and increased blood glucose levels; a finding supported by the study of Hacihamdioglu et al. (25). Moreover, and as expected, fasting glucose/insulin ratio showed continuously significant increases in association with worsening β-cell function.

In Group III patients, for a β-cell function more than 80%, both fasting blood glucose and CRP levels were significantly lower and fasting insulin was higher while IR was intermediate between the other two groups. Fasting glucose/insulin was <7, suggesting abnormal (lower) insulin sensitivity in these patients (20). In addition, we noted that 45% of these children were surgical patients, whereas in the other two groups all the patients were medical patients suggesting that surgery may have less impact on pancreatic β-cell function related to the mild postsurgical inflammatory response (low CRP). A decrease in insulin concentrations after the induction of anesthesia followed by a stress-induced catabolic hyperglycemia [adrenocorticotropic hormone (ACTH) and cortisol-induced] has been described, but the effects are short-lived with normalization within 24–48 h after surgery (26).

Interestingly, we found significantly higher levels of CRP in patients with lower β-cell function. This may be an indication of inflammatory injury of β cells in the ICU setting. In support of this, the experimental work of Chan et al. (27) suggested that CRP-activated NADPH-oxidase redox signaling promoted proinflammatory cytokine production resulting in β-cell damage in this state of inflammation.

In the present study, the mortality rates in patients with HOMA-β% < 40%, in patients with HOMA-β% from 40 to 80, and the patients with HOMA-β% > 80% were 26.3, 25.0, and 4.5%, respectively, but the differences were not statistically significant. However, when the patients were divided into two categories (those with <80% vs. those > 80.0%), the mortality rates were 26.1 vs. 4.5% (*p* = 0.035). In logistic regression, HOMA-β% < 80% and high PRISM III score were the only significant predictors of mortality in a multivariate analysis. On the other hand, an ROC analysis suggests a cutoff value of HOMA-β% of 22 as being 78.9% sensitivity for the prediction of mortality.

The association between β-cell dysfunction and mortality was reported in the study of Das et al. (12) who found that, among a heterogenous group of ICU children and adults, non-survivors had significantly higher levels of β-cell dysfunction. In contrast, the study of El-Sherbini et al. (22) that evaluated the incidence and etiology of stress hyperglycemia in 60 critically ill children found no significant difference between HOMA-β% in survivors and non-survivors.

In our study, HOMA-IR was significantly higher in children with higher BMI Z-score. An increase in BMI has been linked with IR in obese children and adolescents (28).

In the HOMA model, the relationship between basal insulin and glucose levels is described in the context of IR and β-cell function. Individuals with normal blood glucose might have both 100% β-cell function and 100% insulin sensitivity. On the other hand, thin and fit individuals might enjoy high insulin sensitivity (e.g., 200%) necessitating the use of lower (in this case 50%) β-cell function (21). In our study, we observed that Group 1 patients with IR > 1 managed to maintain normoglycemia by increasing the percentage of β-cell function. This finding underpins the importance of viewing IR and β-cell function in tandem. It also suggests that the ability of the body to respond to stress-induced IR by increasing β-cell function is a good prognostic sign.

It is not possible to predict which critically ill children admitted to ICU will develop stress hyperglycemia, identifying those patients who have diminished β-cell function as opposed to IR may direct treatment plans.

The HOMA model studied in this work has the advantage of requiring only a single blood sample for assessing insulin and glucose level, which could be applicable in a developing country with limited resources. However, we acknowledge the following limitations (29): it is somewhat less accurate than the newer HOMA-2 model, it does not differentiate between peripheral and hepatic IR and does not take into account either the increase in insulin secretion that may occur in response to hyperglycemia or renal glucose losses as does the newer model and is more in line with older insulin assays. Our conclusions are also limited by small numbers (especially in some of the patient categories), a lack of serial assessment of β-cell function during PICU stay as well as long-term follow-up of the patients. In the PICU setting, the physiological changes in the critically ill child are rapid, and a single measurement for β-cell function can be misleading. In addition, follow-up would shed a light on the nature and characteristics of the condition. A few studies have demonstrated that adult patients with stress hyperglycemia are at subsequent risk for type-2 diabetes (29).

## Conclusions and Recommendations

Pancreatic β-cell dysfunction (with or without IR) resulting in stress-induced hyperglycemia is related to morbidity and mortality, and its measurement in conjunction with PRISM scores may play a role in predicting poor outcome in high-risk patients. However, it is important to recognize that mortality prediction and association are complex phenomena. The cause of morbidity/mortality is often multifactorial. Long-term follow-up of these patients to see who will demonstrate pancreatic β-cell recovery and who may develop diabetes is an important consideration. In addition, future research into ideal target levels for blood glucose in the ICU setting is needed.

## Data Availability Statement

The raw data supporting the conclusions of this article will be made available by the authors, without undue reservation.

## Ethics Statement

The studies involving human participants were reviewed and approved by the scientific research ethics committee, Faculty of Medicine, Fayoum University, Egypt (R107), in session No 68. Written informed consent to participate in this study was provided by the participants' legal guardian/next of kin.

## Author Contributions

SM, NB, and RY did the research design. SM and NB wrote the manuscript. HAA revised the results and did the statistics. HMA and NK did the laboratory work. RY enrolled the patients and collected the data. All the authors revised the manuscript and approved it.

## Conflict of Interest

The authors declare that the research was conducted in the absence of any commercial or financial relationships that could be construed as a potential conflict of interest.
